# Antibody–drug conjugates as novel anti-cancer chemotherapeutics

**DOI:** 10.1042/BSR20150089

**Published:** 2015-07-14

**Authors:** Christina Peters, Stuart Brown

**Affiliations:** * School of Life Sciences, Nottingham Medical School, Queen's Medical Centre, Nottingham NG7 2UH, U.K.

**Keywords:** antibody–drug conjugates, brentuximab vedotin, cancer, chemotherapy, monoclonal antibodies, trastuzumab emtansine

## Abstract

Over the past couple of decades, antibody–drug conjugates (ADCs) have revolutionized the field of cancer chemotherapy. Unlike conventional treatments that damage healthy tissues upon dose escalation, ADCs utilize monoclonal antibodies (mAbs) to specifically bind tumour-associated target antigens and deliver a highly potent cytotoxic agent. The synergistic combination of mAbs conjugated to small-molecule chemotherapeutics, via a stable linker, has given rise to an extremely efficacious class of anti-cancer drugs with an already large and rapidly growing clinical pipeline. The primary objective of this paper is to review current knowledge and latest developments in the field of ADCs. Upon intravenous administration, ADCs bind to their target antigens and are internalized through receptor-mediated endocytosis. This facilitates the subsequent release of the cytotoxin, which eventually leads to apoptotic cell death of the cancer cell. The three components of ADCs (mAb, linker and cytotoxin) affect the efficacy and toxicity of the conjugate. Optimizing each one, while enhancing the functionality of the ADC as a whole, has been one of the major considerations of ADC design and development. In addition to these, the choice of clinically relevant targets and the position and number of linkages have also been the key determinants of ADC efficacy. The only marketed ADCs, brentuximab vedotin and trastuzumab emtansine (T-DM1), have demonstrated their use against both haematological and solid malignancies respectively. The success of future ADCs relies on improving target selection, increasing cytotoxin potency, developing innovative linkers and overcoming drug resistance. As more research is conducted to tackle these issues, ADCs are likely to become part of the future of targeted cancer therapeutics.

## Abstract (precis)

Antibody drug conjugates (ADCs) are an efficacious class of anti-cancer drugs that comprise monoclonal antibodies (mAbs) conjugated to small-molecule cytotoxic agent via a stable linker. This review summarizes the current knowledge and developments in the field of ADCs.

## INTRODUCTION

The advent of modern-day cancer chemotherapy dates back to the mid-1900s when a chemical warfare agent known as nitrogen mustard was seen to destroy the bone marrow and lymph tissue of exposed individuals [1]. In the following years, nitrogen mustard, along with numerous other alkylating agents [2] took centre stage in the treatment of various haematological malignancies including leukaemia, lymphoma, Hodgkin's disease and multiple myeloma. Several other serendipitous observations [3] lead to the development of the first primitive classes of cytotoxins (Figure 1). Despite vast progress in the field of cancer chemotherapy, small-molecule cancer drugs (although highly potent) continue to be plagued with the problems of non-specific toxicity (as a result of targeting all rapidly dividing cells), narrow therapeutic windows [4] and increasing resistance rates [5]. These concerns emphasize the need to move away from conventional cancer treatments and explore new ways to tackle the ever-present disease.

**Figure 1:**
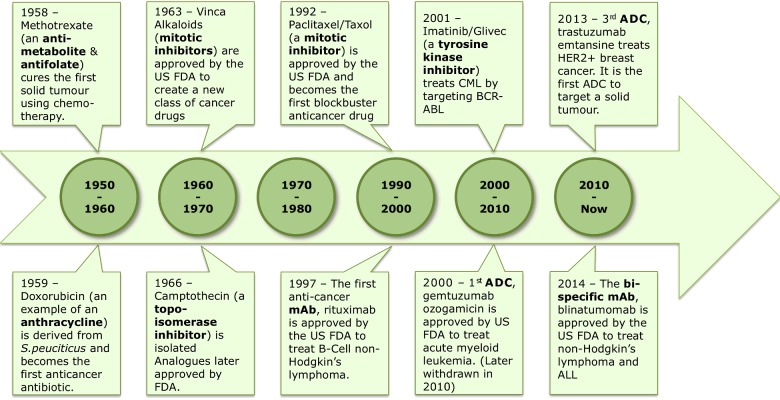
Evolution of chemotherapeutic drugs [6]

In recent years, enhanced understanding of cancer biology has shifted the focus of cancer treatment from traditional chemotherapy to targeted cancer therapies that take advantage of the differentiating features of tumour cells to provide a framework for drug development. These distinctive features, collectively known as the ‘hallmarks of cancer’ [7,8] enable tumour cells to survive, multiply and metastasize using a variety of mechanisms including activation of self-sufficient growth signals, evasion of anti-growth signals, evasion of apoptosis and induction of angiogenesis. Currently approved targeted therapies counteract these and provide safer and more efficacious alternatives to traditional chemotherapy.

Cancer cells differ from normal cells due to genomic mutations in oncogenes and/or tumour suppressor genes [9]. Once the integrity of the genome is compromised, cells are more likely to develop additional genetic faults, some of which may give rise to tumour-specific antigens (found only on the surface of tumour cells) or tumour-associated antigens (overexpressed on tumour cells, but also present on normal cells) [10]. Ongoing research has found that several human cancers express unique tumour-specific or tumour-associated cell surface antigens [11] which are of great value as targets for large molecule, monoclonal antibody (mAb)-based therapy.

The use of antibodies as ‘magic bullets’ to treat disease was first proposed more than 100 years ago by the founder of chemotherapy, Paul Ehrlich [12]. Due to several challenges in the development of human antibodies, it was only in 1997 that the US FDA (Food and Drug Administration) approved the first anti-cancer antibody, rituximab, for the treatment of B-cell non-Hodgkin's lymphoma [13]. Early mAbs were based on murine or chimeric antibodies that were modified to target human antigens. As these were non-human antibodies, they evoked a strong immune response that prevented the treatment from being successful. The large size of the mAbs also proved to be problematic as it resulted in reduced tumour penetration [14] and poor therapeutic effect. Since then, several advances in antibody engineering [15,16] have optimized pharmacokinetics and effector function while reducing immunogenicity. This has resulted in a significant increase in the development of antibody-based drugs [17,18] against cancer.

mAbs exert their therapeutic effect [19] by binding to tumour-specific or tumour-associated cell surface antigens. Once bound, the mAb kills the tumour cell by one or more of the following mechanisms; (i) abrogation of tumour cell signalling, resulting in apoptosis (ii) modulation of T-cell function through antibody-dependent cellular cytotoxicity (ADCC), complement-dependent cytotoxicity (CDC) or complement-dependent cell-mediated cytotoxicity (CDCC) and (iii) exertion of inhibitory effects on tumour vasculature and stroma [20,21]. Despite these various cell-killing mechanisms, most mAbs display insufficient cytotoxic activity [22]. Current efforts in cancer treatment have therefore focused on combining the selectivity of mAbs with the potency of chemotherapeutic small molecules, giving rise to an entirely new class of anti-cancer drugs known as antibody-drug conjugates (ADCs) [23].

ADCs are tripartite drugs comprising a tumour-specific mAb conjugated to a potent cytotoxin via a stable linker (Figure 2). The three components of the ADC together give rise to a powerful oncolytic agent capable of delivering normally intolerable cytotoxins directly to cancer cells, which then internalize and release the cell-destroying drugs [24]. Although the concept of combining a mAb with a cytotoxic drug is fairly old, significant improvements have been made since the first generation of ADCs. Early ADCs (based on mAbs that did not undergo internalization) were engineered to release their active drug once tumour-specific enzymes (such as matrix metalloproteinases) or tumour-specific environments (such as a lower pH) cleaved the linker component of the ADC [25]. These non-internalizing ADCs failed to significantly improve drug specificity and therefore did little to decrease toxicity [26]. Since then, the development of internalizing conjugates has increased their therapeutic potential. The targeted nature of ADCs allows for increased drug potency coupled with limited systemic exposure. Together, these features clinically manifest themselves as fewer side effects and a wider therapeutic window. In addition, the internalization of the ADC reduces drug resistance arising from P-glycoprotein (P-gp)-mediated efflux mechanisms [27]. ADCs are therefore capable of bypassing major issues in both traditional and targeted chemotherapy.

**Figure 2:**
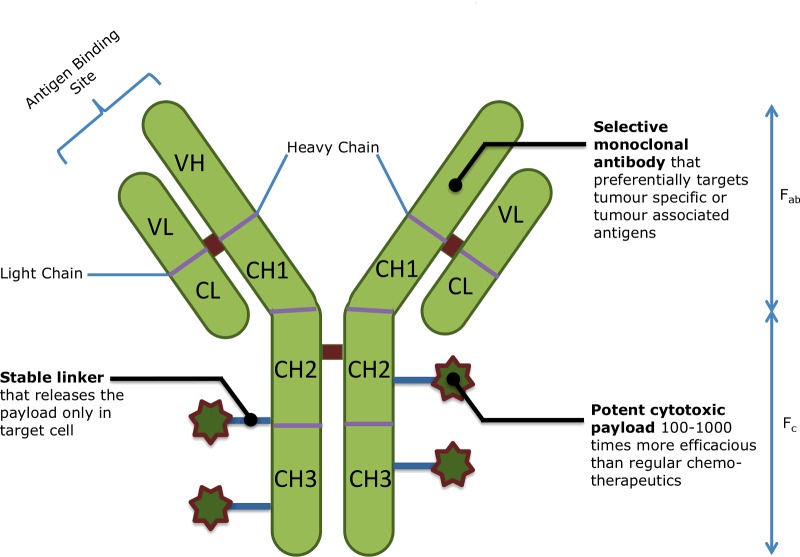
Structure of an ADC [28]

Although the concept of ADCs is theoretically simple, it is difficult to combine its various components into an optimized and functional therapeutic agent. To date, three ADCs have gained entry into the market, of which only two remain [29]. The first ADC to obtain FDA approval was gemtuzumab ozogamicin (Mylotarg®), marketed by Wyeth (now Pfizer), for the treatment of relapsed acute myeloid leukaemia (AML) [30]. In 2010, a decade after its approval, gemtuzumab ozogamicin was withdrawn from the market when a clinical trial showed that it had little benefit over conventional cancer therapies [31] and was associated with serious hepatotoxicity [32]. This could have been due to the fact that the linker technology used was not stable enough to prevent the drug from being released in the bloodstream [33]. The two ADCs currently in the market are brentuximab vedotin (Adcetris® by Seattle Genetics) and trastuzumab emtansine (T-DM1; Kadcyla® by Genentech). The former is the first of the two ADCs to be approved and is currently being used to treat patients with relapsed or refractory Hodgkin's lymphoma or those with relapsed or refractory systemic anaplastic large cell lymphoma [34]. The more recent approval of T-DM1, in 2014, [35] for use against breast cancer, proved that ADCs were capable of targeting solid tumours in addition to haematological malignancies. Although there are only 2 ADCs currently in the market, there are more than 30 ADCs being developed to target a wide range of blood cancers and solid tumours. In contrast with small molecules that have a limited choice of drug targets, the diverse range of ADC targets results in a robust drug pipeline with minimal overlap between different pharmaceutical companies [36]. With the recent FDA approvals of brentuximab vedotin and T-DM1, there has been an increase in research investigating the use of ADCs in the treatment of cancer. Recent reviews on ADCs have summarized the preclinical and clinical advances made in the field and have discussed key characteristics of marketed ADCs [37–39]. They have also provided an overview of linker properties, drug releasing strategies and viable targets for the design of ADCs [40,41]. The primary objective of this article is to produce an updated, comprehensive review of the current knowledge in these areas, based on the developments in the last few years.

## MECHANISM OF ACTION OF ADCs

When designing an ideal ADC, it is essential to understand the mechanism of action in order to identify the desired features of each of its three components. An ideal ADC is one that retains the selectivity and killing capacity of a mAb while still being able to release the cytotoxic drug in quantities large enough to kill tumour cells. Each of the steps involved in the mechanism of action is associated with unique challenges [33] that complicate the design of ADCs. These are illustrated in Figure 3.

**Figure 3:**
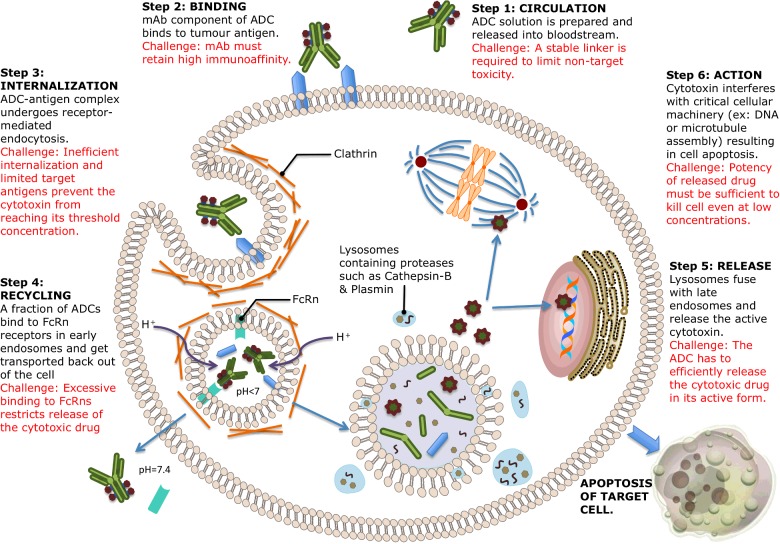
Mechanism of action of ADCs

ADCs are administered intravenously in order to prevent the mAb from being destroyed by gastric acids and proteolytic enzymes. The mAb component of the ADC enables it to circulate in the bloodstream until it finds and binds to tumour-specific (or tumour-associated) cell surface antigens present on target cancer cells. In the interest of preventing unwarranted release of the cytotoxin and maximizing drug delivery to cancer cells, an ideal linker would not only have to be stable in the bloodstream but also capable of releasing the active form of the cytotoxic drug when required [42].

Once the mAb component of the ADC is bound to its target antigen, the ADC–antigen complex should theoretically be internalized via receptor-mediated endocytosis. The internalization process finishes with the formation of a clathrin-coated early endosome containing the ADC–antigen complex [43]. An influx of H^+^ ions into the endosome results in an acidic environment that facilitates the interaction between the mAb component of a fraction of ADCs and human neonatal Fc receptors (FcRns). The bound ADCs are transported outside the cell, where the physiological pH of 7.4 enables the release of the ADC from the FcRn [44]. This mechanism acts as a buffer for preventing the death of healthy cells in the case of ADC mis-delivery. Excessive binding of ADCs to tumour cell FcRns might however restrict the release of the cytotoxic drug and prevent the ADC from taking effect [45]. FcRn expression is primarily within the endosomes of endothelial cells.

ADCs that remain in the endosome without binding to FcRn receptors form the late endosome. These subsequently undergo lysosomal degradation, allowing the release of the cytotoxic drug into the cytoplasm. At this stage, it is crucial to ensure that a sufficient (i.e. threshold) concentration of the drug is present within the cancer cell for its destruction to be guaranteed. This is however complicated in practice by the facts that cell-surface antigens are often quite limited and the process of internalization, rather inefficient [46]. Assuming all the steps involved in the mechanism of ADC action have an efficiency of 50%, only 1%–2% of the administered drug will reach tumour cells [47]. This makes the choice of cytotoxin particularly important, as it is required to be highly efficacious at very low concentrations. Drugs that are usually unsuitable for normal chemotherapy (due to excessive toxicity) are therefore a necessary component of ADCs.

Different classes of cytotoxic drugs result in cell death using various mechanisms [39]. The common element between all the classes is interference with critical cell functioning and, as a consequence, either direct killing of the cell or inducion of apoptosis. As targeted cancer cells die, there is potential for the active cytotoxic drug to kill neighbouring tumour cells and the supporting stromal tissue. The design of ADCs with respect to the choice of target, mAb, linker and cytotoxin are all very important determinants of whether or not the threshold concentration of the cytotoxic drug is reached within the tumour cell. These factors therefore determine the overall success of an ADC.

### Target Antigen Selection

Successful development of an ADC is dependent on the selection of an appropriate target antigen to which the mAb component of the ADC can bind. Aside from being tumour-specific or tumour-associated, cell surface antigens should also undergo efficient internalization, have high levels of expression and possess high penetrance, a characteristic whereby a large percentage of tumour samples test positive for the presence of the antigen. The target of gemtuzumab ozogamicin, an ADC previously used against AML, was cluster designation 33 (CD33), a transmembrane cell-surface glycoprotein expressed on the surface of mature and immature myeloid cells. CD33 has extremely high penetrance with 90%–95% of all AML patients testing positive for the antigen [48]. With regard to tumour specificity and sensitivity however, CD33 performed rather poorly as it was found to have only low levels of expression on not only on mature and immature myeloid cells but also erythroid cells, megakaryocytes and multipotent progenitor cells [49,50]. Current ADCs aim to execute their therapeutic action by identifying target antigens that fulfil all four of their requirements.

Although tumour-specific antigens are ideal, most ADC targets tend to be tumour-associated. The expression of such antigens should be kept to a minimum on healthy tissue cells, [51–53] unless these cells are insensitive to drug action. Examples of target antigens that are expressed in both normal tissues as well as cancer tissues include the prostate-specific membrane antigen (PSMA) and the HER2 (human epidermal growth factor receptor 2) receptor [38]. In the case of PSMA, it is expressed only within the cytoplasm of healthy prostate tissue and therefore remains unaffected by ADCs that target extracellular PSMA in prostate cancer cells [54]. On the other hand, ADCs targeting the HER2 receptor require the antigen to be highly overexpressed in breast cancer cells in comparison with healthy cells in order for the normal tissue to remain unaffected by the drug.

The level of antigen expression required for effective ADC activity varies depending on the antigen in question. As mentioned earlier, HER2 antigens on breast cancer cells are required to be highly overexpressed for effective targeting [55]. In contrast, CD19 antigens targeted in B-cell lymphoma may be limited to as few as 30000 per cell in order to elicit ADC activity [56]. There is, however, a minimum requirement of approximately 10000 antigens per cell, in order to ensure the delivery of lethal quantities of the cytotoxic drug [57]. The variability in the prerequisite number of targets emphasizes the need to use preclinical tumour models that are similar to human tumours with respect to antigen expression levels. An added complication arises from the fact that the initial estimate of antigen expression does not stay constant, but instead varies during the course of the treatment [58].

In addition to specific and sufficient expression, an optimal target antigen should also incite effective ADC internalization [51]. The rate and efficiency of internalization depends on the type of target and the choice of cytotoxin. Some targets internalize frequently regardless of ligand binding, whereas others reside permanently on the cell surface. Although it was initially thought that ADCs targeting non-internalizing antigens would have poor efficacy [59], a study using rituximab bound to doxorubicin/auristatin showed it underwent efficient internalization despite binding to the non-internalizing antigen CD20. This was however, not the case when the same mAb was conjugated to a different cytotoxic drug, calicheamicin [39]. The internalization efficiency of some antigens also depends on the specific epitope that binds to the mAb, as this leads to varying levels of antigen–antibody affinity [60,61].

Some ADCs are capable of eliciting antigen-mediated anti-tumour activity in addition to the cytotoxic activity arising from the conjugated drug. This is possible when the target antigen has a biological role in cancer pathways that is subsequently inhibited by the binding of an antibody. The most commonly cited example of an ADC with proven anti-tumour activity resulting from the target antigen is T-DM1 [62]. Trastuzumab is a humanized antibody that targets the transmembrane tyrosine kinase receptor, HER2 [a member of the EGFR (epidermal growth factor receptor) family] that is commonly overexpressed in a quarter of all breast cancers [63]. Unlike most other receptors, HER2 has no known endogenous ligand but is instead activated by forming homo or heterodimers with other members of the EGFR family [64]. Once HER2 is activated, downstream effector molecules initiate intracellular (tumour-inducing) signalling pathways such as the phosphoinositide 3 kinase (PI3K) pathway (which prevents cellular apoptosis) [65] and the mitogen-activated protein kinase (MAPK) pathway (which promotes cell proliferation) [66].

### Types of Tumour-Associated Antigens

Although cell-surface proteins are the most commonly used targets for ADCs, there are various other categories of tumour-associated antigens including glycoproteins, proteins of the extracellular matrix and aberrant gangliosides on the tumour cell surface [67,68]. Apart from antigens found on tumour cells, there is a growing interest in targeting antigens present on tissues that support the growth and spread of tumour cells such as neovasculature or extracellular stromal tissue [69–73]. This is particularly attractive as these tissues have a stable genome unlike cancer cells and are therefore less likely to undergo somatic mutations, reducing the risk of mutation-mediated drug resistance. Instead, non-tumour tissue targets differentiate themselves from healthy tissue by being in an undeveloped state as a result of their rapid formation and turnover rate [74]. ADCs that bind to neovasculature destroy the tumour blood supply and cause tumour cell death via nutrient deprivation and hypoxia [75,76]. Potential targets in tumour vasculature include vascular endothelial growth factor (VEGF) and its receptors integrin and endoglin. ADCs that target and destroy extracellular stromal tissue cause tumour cell death by reducing the concentration of growth factors produced by stroma. Similar to tumour vasculature, these growth factors are critical in promoting tumour cell survival [77]. Examples of anti-tumour stromal targets include fibroblast activation protein (FAP) and protein tyrosine kinase 7 (Ptk 7), a pseudokinase enzyme commonly found on many cancer and stromal cells [78]. Since all tumours depend on angiogenesis and stromal factors for their survival and growth, ADCs that target such tissues have a wider efficacy.

### Choosing Monoclonal Antibodies

mAbs allow ADCs to have high target-specificity, target-affinity and prolonged drug exposure at the tumour site. Based on these features, antibody selection should ideally ensure minimal cross-reactivity with healthy tissues, sub-nanomolar affinity to the target antigen and a long pharmacokinetic half-life combined with minimal immunogenicity [79]. Over time, these features result in the accumulation of the ADC at the tumour site and allow it to have an increased therapeutic effect. In addition, it is beneficial for the mAb to possess intrinsic anti-tumour activity resulting from either direct modulation of the biological activity of the target antigen (e.g., anti-HER2 mAbs) [80] and/or via immune effector functions such as ADCC, CDC and CDCC [81]. When constructing the ideal ADC, it is important to maximally preserve the favourable properties of the mAb.

With regard to tumour specificity and target affinity, it is important to choose a mAb that does not lose these features through non-specific binding to normal cells. Apart from being toxic to healthy tissue, an antibody lacking tumour specificity may be eliminated from the circulation due to immunogenicity, leading to sub-optimal target exposure and decreased therapeutic effect [82]. The complementarity-determining regions of an antibody (i.e. the antigen-binding sites) should have extremely high (preferably sub-nanomolar) target affinity in order to guarantee efficient internalization [83].

The immunogenicity of an ADC is one of the major determinants of its circulatory half-life. Early ADCs made use of murine mAbs that evoked a strong, acute immune response in humans that resulted in the rapid formation of human anti-mouse antibodies within 2 weeks of a single dose [84]. Since then, murine mAbs have been replaced with chimeric IgG antibodies that have a human constant region and a murine variable region [85]. Another alternative is the use of humanized IgG antibodies that have a completely human variable sequence except for the portion responsible for antibody-antigen complementarity [86]. Most ADCs that are currently in use or in clinical development employ either humanized or fully human antibodies [19]. Brentuximab vedotin (an anti-CD30 ADC) [34,87] and BT062 (an anti-CD138 ADC) both incorporate chimeric mAbs [88].

A major benefit of using mAbs and mAb-based drugs, such as ADCs, is their favourable pharmacokinetics with respect to distribution, metabolism and elimination in comparison with small-molecule cancer therapies. Once mAbs are administered into the bloodstream, they can distribute into cancer tissue either via extravasation through pores in the endothelium or via pinocytosis through endothelial cells following a diffusion gradient [89]. The distribution of the ADC (and hence the cytotoxic drug) into tumour tissues is limited by the size of the antibody, which represents more than 90% of the mass of an ADC [90]. However, unlike normal blood vessels that have a monolayer of endothelial cells forming tight junctions with one another, tumour endothelium is characterized by excessive branching and sprouting, resulting in a leaky monolayer [91]. The large size of ADCs therefore does not necessarily restrict their distribution into tumour tissue but minimizes their distribution into metabolizing and eliminating organs such as the liver, intestines, muscle and skin, thereby extending their half-life [92–94]. An additional mechanism by which ADC half-life is prolonged is through the binding of the Fc portion of humanized IgG antibodies to receptors within endosomal vesicles of endothelial cells known as human FcRns [95]. The resulting FcRn–ADC complex can either be transported back into systemic circulation or into the interstitial fluid surrounding tumour cells. FcRns therefore act as a useful reservoir for ADCs following the pinocytosis of the mAb into the endothelial cell. As the concentration of FcRns within endothelial cells is very high [96] and probably exceeds that of the internalized ADCs, their transport out of the cell is extremely efficient and prevents the ADC from causing unwanted endothelial cell death. Extensive research is being conducted to improve the binding of ADCs to endothelial FcRn receptors and thereby prolong their circulatory half-life [97].

The anti-tumour activity of mAbs may occur by both direct and indirect mechanisms [20]. Certain mAbs such as trastuzumab or rituximab exert direct cytotoxic effects by blocking the biological signalling activities of tumour antigens associated with cell-function and proliferation [98,99]. Other antibodies have indirect effects by promoting natural anti-tumour immune response mechanisms such as ADCC, CDC or CDCC (Figure 4). In ADCC, immune effector cells, such as macrophages and natural killer (NK) cells, bind to the CH3 region of IgG mAbs via their FcγRIIIa receptors [100]. Once bound, the immune cells become activated and mediate tumour cell killing via phagocytosis by macrophages or via the release of toxic granules from NK cells [101]. In CDC, the binding of a mAb to its target antigen on tumour cells triggers the classical complement pathway involving up to 30 circulating plasma proteins. The complement pathway begins with the binding of C1q to the second constant heavy chain (CH2) region of mAbs, resulting in the activation of a proteolytic cascade that terminates with the formation of a membrane attack complex. This complex results in tumour cell death via the action of pore-forming structures that release cellular contents [102]. In CDCC, another protein formed during the complement cascade, C3b, acts as an opsonin and interacts with C3b receptors present on NK cells and macrophages to facilitate tumour cell lysis. As all the effector functions of mAbs, require the binding of the constant region to various receptors, they work best when the target antigen undergoes limited internalization.

**Figure 4:**
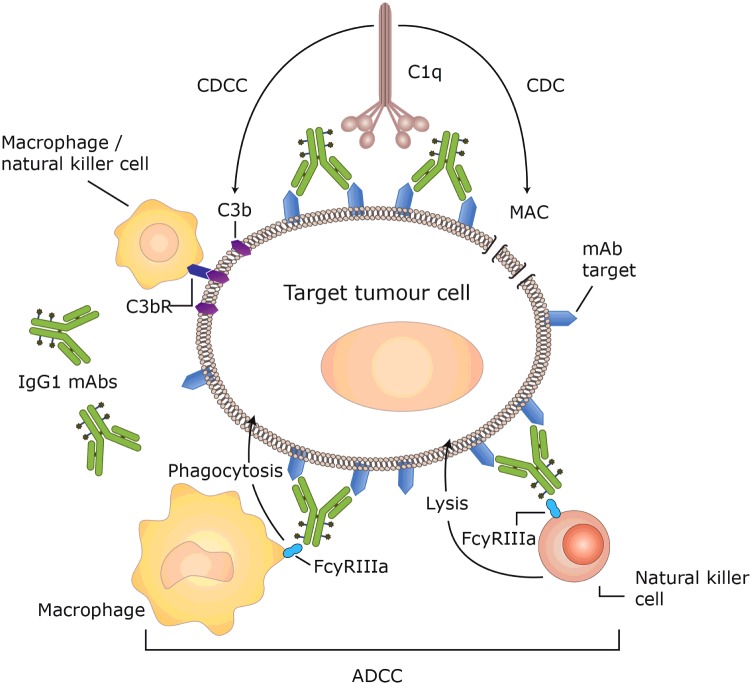
Mechanism of ADCC, CDCC and CDC [103]

The strength of the various immune effector functions varies depending on the specific isotype of monoclonal IgG antibodies used in the ADC. The effects of ADCC and CDC are much stronger in human IgG1 and IgG3 isotypes in comparison with IgG4 and IgG2 mAbs [104,105]. Although efficient at tumour cell lysis, the short circulation half-life of IgG3 antibodies compared with IgGs 1, 2 and 4 makes them a poor choice for use in ADCs [104,106]. IgG4 antibodies are also not preferred due to their tendency to exchange one-half of themselves with another IgG4 antibody with the possibility to form a new hybrid IgG4 mAb. In order to overcome this, the CH3 region of IgG4s, responsible for the formation of hybrid antibodies, can be replaced with the CH3 region of IgG1 mAbs [107]. IgG2 antibodies are favoured for therapeutic use due to their tendency to form covalent dimers that aid antibody–antibody associations. These associations have the potential to enhance the affinity and/or internalization of the mAb but may also cause the ADC to aggregate and become ineffective [108]. The difference in the extent of immune effector function is evident in previously approved ADCs that show substantial variation with regard to intrinsic anti-tumour activity. Gemtuzumab ozogamicin, for example, uses an IgG4 mAb that lacks any effector activity, [32] whereas brentuximab vedotin that is based on an IgG1 sub-type shows much better (although still modest) ADCC action [109].

Enhanced antibody functionality may be achieved by employing bispecific antibodies engineered to comprise two different antigen-binding sites capable of recognizing two different epitopes. These can be on the same antigen or on different antigens, usually on the surface of two different cells [110]. The first therapeutic bispecific antibody, catumaxomab, was approved in Europe in 2009 and targets EpCAM antigens (on tumour cells) and CD3 (on T-cells). In 2014, the US FDA gave accelerated approval for a bispecific antibody blinatumomab, designed based on bispecific T-cell engager (BiTE) immunotherapy. Blinatumomab binds CD19 on the surface of B-cell lymphoblasts and CD3 on the surface of T-cells, thus bringing them together and enabling T-cell mediated killing of the tumour cells. More bispecific antibodies are in clinical development for oncology, autoimmune and infectious-disease indications [111]. A variety of bispecific antibodies with tumour-specific antigens such as HER2/neu, CEA (carcinoembryonic antigen) and CD30 have all been clinically validated. The design of bispecific antibodies is however complicated by the need to engineer multiple antigen-binding domains into a single construct.

Although mAbs can now be engineered to have increased circulation half-lives and improved immune effector functions [112,113], there is limited clinical data regarding the use of such optimized antibodies for enhanced ADC function. The notion that ADCs with high target affinity possess high cytotoxic capacity is, for example, yet to be demonstrated in preclinical models [114] or in the clinic. Goldmacher and Kovtun [38] have hypothesized that such a correlation may in fact not exist, as ADCs with high target affinity may rapidly bind to perivascular regions of the tumour rather than bind uniformly to all tumour cells [115,116]. Similarly, the assumption that ADCC and CDC contribute to the anti-tumour activity of ADCs [104,105] might prove to be incorrect as these effector functions are generally weak and compete with ADC internalization. In fact, IgG2 and IgG4 isotypes of antibodies that have poor immune effector function for naked mAbs were the preferred antibodies for use in certain ADCs [30,117,118]. The benefit of using fully human antibodies as opposed to chimeric and humanized versions has also not been demonstrated in clinical trials, as patients with advanced cancers lack the ability to produce antibodies against therapeutic mAbs [90]. These poorly established criteria for antibody selection and their impact on ADC activity make it difficult to design an optimized mAb.

### Linkers

Linker chemistry is an important determinant of the safety, specificity, potency and activity of ADCs. Linkers are designed to be stable in the blood stream (to conform to the increased circulation time of mAbs) and labile at the cancer site to allow rapid release of the cytotoxic drug [119]. There are various parameters that are taken into consideration when designing the ideal linker. These include the cleavability of the linker and the position and mechanism of linkage (i.e. conjugation chemistry) [42]. Linkers are traditionally classified based on the first parameter, i.e. the mechanism by which they release the cytotoxin [51]. Existing linkers belong to either one of two broad linker designs: cleavable or non-cleavable linkers.

Cleavable linkers exploit the differences between conditions in the blood stream and the cytoplasmic conditions within cancer cells [120]. The change in environment once the ADC–antigen complex is internalized, triggers cleavage of the linker and release of the active drug, effectively targeting toxicity to cancer cells. There are three types of cleavable linkers: hydrazone, disulfide and peptide linkers, each of which responds to different cancer-specific intracellular conditions. Hydrazone linkers were among the first to be developed and depend on the low pH within lysosomes to undergo acid hydrolysis and release the cytotoxic drug [121]. Gemtuzumab ozogamicin is an example of an ADC that used acid-labile hydrazone linkers. Unfortunately, the stability of such linkers came under scrutiny [33] when results from a clinical trial showed that gemtuzumab ozogamicin was associated with significantly higher toxicity in comparison with standard therapy [31]. An alternative to hydrazone linkers is the use of disulfide linkers. These take advantage of elevated concentrations of thiol molecules (e.g., glutathione) within cancer cells. Thiol molecules are especially high in tumours, as they are involved in promoting cell-survival and tumour growth and are produced during cell-stress conditions such as hypoxia [122]. The third type of cleavable linker is the enzyme labile or peptide linker. In comparison with hydrazone and disulfide linkers, peptide linkers offer improved control of drug release by attaching the cytotoxic drug to the mAb via a dipeptide linkage [123]. The proteases required to break the peptide bond are only active in low pH environments, making it highly unlikely that the cytotoxic drug is released in the pH-neutral environment of the blood. Instead, the dipeptide linkage is cleaved in the acidic environment within lysosomes by lysosomal proteases, such as cathepsin-B and plasmin [124]. Brentuximab vedotin is an example of an ADC that employs a dipeptide linkage consisting of valine and citrulline along with a para-aminobenzylcarbamate spacer molecule that separates the large cytotoxic drug from the mAb [125]. A clinical trial comparing the three cleavable linkers showed that enzyme-labile linkers had lower *in vivo* toxicity as a result of greater specificity, increased stability and a longer half-life compared to hydrazone linkers [126].

In contrast with cleavable linkers that are reliant on distinctive intracellular conditions to release the cytotoxin, non-cleavable linkers depend solely on the process of lysosomal degradation following ADC-antigen internalization. Protease enzymes within the lysosome breakdown the protein structure of the mAb, leaving behind a single amino acid (usually a lysine or a cysteine) still attached to the linker and cytotoxin. The resulting amino acid–linker–cytotoxin complex is released into the cytoplasm and subsequently becomes the active drug. In comparison with cleavable linkers, non-cleavable linkers were found to have improved stability in the bloodstream allowing ADCs with such linkers to have longer half-lives and pose a reduced risk from side effects while retaining the activity of the cytotoxic drug [127]. This knowledge was reflected in the clinical development of T-DM1, originally designed to have a valine–citrulline dipeptide linker but was instead produced with a non-cleavable thioether linker [128]. Despite the successful use of non-cleavable linkers, it is important to note that their dependence on lysosomal degradation means they can only be used in ADCs targeting antigens that undergo efficient intracellular internalization.

Once an optimal linker for a particular ADC is chosen, it is important to determine the ideal number of drugs to be conjugated to a mAb [i.e., the drug–antibody ratio (DAR)]. Because of potential linker instability and poor ADC internalization, several drugs are required to be linked to each mAb to achieve adequate cytotoxicity. On the other hand, excessive drug conjugation might result in increased clearance and/or immunogenicity as the ADC is more likely to aggregate and be recognized as a damaged, malformed or foreign protein [129]. In a study conducted by Hamblett et al. [130], the *in vivo* efficacy of ADCs containing four drugs per antibody was found to be equivalent to that of ADCs with eight drugs per mAb. Heavily modified ADCs (i.e., those with eight conjugated drugs) were associated with increased toxicity and underwent faster clearance from the circulation. Additionally, it was found that ADCs containing only two drugs per mAb had a much wider therapeutic window and therefore a higher maximum tolerated dose. The study consequently concluded that ADCs loaded with 2–4 drugs per mAb achieved the best balance between slow clearance and maximal potency [130].

A critical aspect of linker function is the presence or absence of a phenomenon known as the bystander effect. This effect refers to target-cell mediated killing of healthy cells neighbouring the tumour cell. The bystander effect is generally caused by cellular efflux of hydrophobic cytotoxic drugs, capable of diffusing out of an antigen-positive target cell and into adjacent antigen-negative healthy cells. Although the bystander effect undermines the target specificity of ADCs, it can be advantageous when tackling solid tumours that lack homogenous expression of the target antigen [131]. However, the bystander effect is only observed with the use of cleavable linkers, as the amino acid-linker-cytotoxin complex formed from the breakdown of non-cleavable linkers is charged and therefore not capable of crossing the hydrophobic lipid bilayer of the target cell [132]. Cleavable linkers are therefore associated with wider efficacy as they can take effect independent of internalization. However, the extent to which they can cause bystander cell death depends largely on conjugation chemistry [133]. Regardless of the extent of cytotoxicity, non-selective ADCs with cleavable linkers are only viable if the bystander effect is restricted to a small number of non-target cells.

### Conjugation Chemistry

In recent years, a great deal of research has been conducted in order to develop novel conjugation techniques for use in future ADCs. Traditionally, cytotoxic drugs have undergone chemical conjugation to mAbs, whereby reactive portions of native amino acids are made to interact and bind a specific part of the linker molecule [134]. Examples of reactive groups include the epsilon-amino end of lysine residues (used in the conjugation of T-DM1) [135] and the thiol side chains present in the partially reduced form of cysteine residues (used in brentuximab vedotin) [136]. As this technique relies on native amino acids, conjugation of the drug is limited by the peptide sequence of the antibody, which therefore restricts control over the number and position of attached cytotoxic drugs. The resulting heterogenous ADC mixture is a major drawback to the chemical conjugation technique as it impacts the toxicity, stability and potency of the ADC.

Heterogeneity, with respect to the number of cytotoxin molecules attached per antibody (Figure 5), results in only a small portion of the prepared ADC solution being therapeutically active. This is because a subset of ADCs will contain too few cytotoxin molecules to retain their cell-killing capacity whereas others will have too many to maintain their stability in the bloodstream [137]. Furthermore, inactive ADCs directly decrease ADC potency by binding the limited number of target antigens available on tumour cells and blocking the binding of therapeutically-active ADCs.

**Figure 5:**
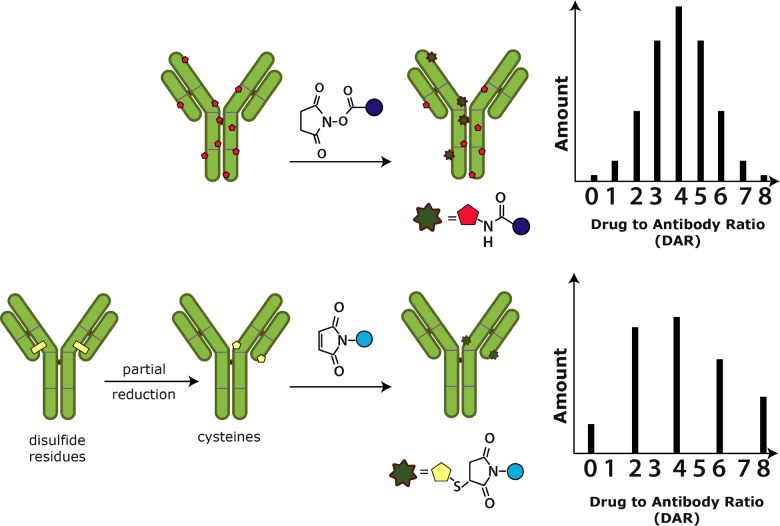
Mechanisms of traditional conjugation with DARs [138]

Heterogeneity, with respect to the position of the cytotoxin on the mAb, plays an important role in the therapeutic effect of ADCs. In an experiment conducted by Shen et al. [139], ADCs with a cytotoxin conjugated at the light chain of the mAb were shown to have significantly higher *in vivo* efficacy compared with heavy chain conjugates.

Currently researched methods for the development of site-specific drug conjugation are based on introducing selectively reactive molecules at specific locations along the mAb. This allows more control over the site and number of drug attachments and can potentially increase the therapeutic window of ADCs, while reducing toxicity and improving pharmacokinetics [140]. The three alternatives to conventional conjugation techniques include conjugation via novel unpaired cysteine residues conjugation via transglutaminases and, finally, conjugation via unnatural amino acids.

Conjugation of novel unpaired cysteine residues to a small portion of the mAb relies on site-directed mutagenesis to introduce a fixed number of engineered cysteines at specific, controlled sites along the mAb [141,142]. This process maintains existing disulfide bonds between native cysteine residues while avoiding the problem of ADC heterogeneity. A study conducted by Junutula et al. [143] showed that T-DM1, when conjugated with unpaired cysteine residues, was as efficacious as traditional T-DM1, albeit with fewer side effects and therefore a wider therapeutic window. This technique is however associated with several drawbacks as the newly introduced, unpaired cysteine residues are at risk of reacting with native cysteine amino acids and creating malformed disulfide bridges that could potentially disrupt the binding capacity of the mAb [144]. There is also the possibility of the unpaired cysteine residues reacting with nearby cysteines on neighbouring mAbs and producing an antibody dimer that could again destabilize the mAb and undermine its functionality [145]. Given these potential problems, this technique is best used when specific sites are identified on the mAb where the new cysteine residues are unlikely to react with other amino acids.

A second method of site-specific drug conjugation uses a microbial transglutaminase enzyme in order to conjugate an amine-containing cytotoxic drug to a mAb modified to have a specific number of glutamine side chains [146,147]. A major advantage of the microbial transglutaminase technique is that it does not recognize pre-existing, native amino acids, [148] but instead interacts with glutamine ‘tag’ sequences that can be incorporated into the mAb via plasmids. This technique allows better control over drug conjugation due to an increased number of possible conjugation sites on the mAb. A study conducted by Strop et al. [149] used microbial transglutaminase to conjugate an EGFR targeting mAb and an anti-M1S1 (chromosome 1, surface marker 1) antibody to the cytotoxic drug monomethyl dolastatin 10. There were several sites of successful conjugation in both conjugates with both of them displaying strong *in vivo* (and *in vitro*) activity. Apart from transglutaminases, other enzymes such as glycotransferases- and formylglycine-generating enzymes have also been investigated for use in a similar manner [150,150].

The final method of drug conjugation is based on the use of non-natural amino acids, such as selenocysteine or acetylphenylalanine [151,152]. These are structurally very similar to their natural amino acid counterparts with only minor differences in functional groups. Selenocysteine, for example, differs from the traditional cysteine residue by substituting a sulfur atom with a selenium atom. Similarly, acetylphenylalanine contains a ketone group that is not present on any of the 20 naturally occurring amino acids [153]. These small differences allow site-specific modification while sparing the pre-existing residues of the mAb. Unnatural amino acids are introduced into the mAb during transcription with the use of stop codons paired to the insertion sequences of selenocysteine or acetylphenylalanine [154]. Once the unnatural amino acid is introduced into the mAb, it is available for conjugation with a suitable cytotoxin, which, in the case of nucleophilic selenocysteine [155] has to be a positively charged molecule. A study that examined the binding of a mutant anti-HER2 mAb containing acetylphenylalanine found that its affinity for its ligand, the HER2 receptor, was comparable to T-DM1 [156]. In addition to the two unnatural amino acids mentioned, there are several others that are currently being developed that may be introduced to the mAb during *in vitro* transcription and translation [157].

### Cytotoxins

As ADCs are most often prepared in an aqueous solution and administered intravenously, it is important that the cytotoxic drug has prolonged stability in such environments to prevent damage to healthy cells and increase the availability of the drug at the tumour site. Similarly, it is important that the molecular structure of the cytotoxin allows for its conjugation to the linker while avoiding immunogenicity, maintaining the internalization rate of the mAb and promoting its anti-tumour effects (i.e., ADCC, CDCC and CDC) [46]. Regardless of the stability of the cytotoxin, only a small portion of the administered ADC reaches the tumour cells. This makes it imperative that the conjugated cytotoxic drug is potent at low concentrations. Typically, the cytotoxins used in ADCs are a 100–1000 times more potent than regular chemotherapeutics and preferably have sub-nanomolar potency [158]. Most classes of cytotoxins act to inhibit cell division and are classified based on their mechanism of action. Since many ADCs utilize cytotoxins that target rapidly dividing cells, there is a decreased risk of unwanted toxicity if the ADC mistakenly delivers the drug to a non-replicating cell. As the cytotoxin is most commonly released in the lysosome following cleavage of the linker molecule, it is important to ensure that the cytotoxin remains stable in low pH environments and has the capacity to move into the cytosolic or nuclear compartments of the cell within which it takes effect. The choice of the specific cytotoxin to be used in an ADC depends on its mechanism of action and the type of cancer.

First generation ADCs made use of early cytotoxins such as the anthracycline, doxorubicin or the anti-metabolite/antifolate agent, methotrexate [159,160]. Although these cytotoxins worked well when administered as standard chemotherapy, they proved to have insufficient potency at low concentrations when conjugated to a mAb [161]. Current cytotoxins have far greater potency and can be divided into three main groups: auristatins, maytansines and calicheamicins. The former two both target rapidly dividing cells by interfering with different parts of the cell cycle [162,163] whereas calicheamicins, along with the less commonly used groups of cytotoxins: duocarymycins and pyrrolobenzodiazepine (PBD) dimers, all induce DNA damage [164]. Regardless of the cell-killing mechanism, all five categories of cytotoxins result in cancer cell death by induction of apoptosis [165].

The auristatins, monomethyl auristatin E (MMAE) and monomethyl auristatin F (MMAF) are specific types of mitotic inhibitors that share their mechanism of action with the traditionally used taxane chemotherapeutics. Auristatins interfere with the formation of microtubules by binding to the β-subunit of α-β tubulin dimers in the cytoplasm. The drug subsequently takes effect by preventing the hydrolysis of GTP molecules on the β-subunit, causing continuous and excessive growth of microtubules (Figure 6). As the microtubules lose their capacity to shorten and separate sister chromatids during anaphase, the cell becomes frozen in the metaphase portion of mitosis [166]. MMAE is used in brentuximab vedotin as well as several other ADCs currently under clinical development [167,168]. Since MMAE is hydrophobic, it can easily diffuse out of the target cell and mediate the killing of nearby bystander cells [169]. This might be a potential drawback for the use of MMAE in ADCs targeting non-solid haematological cancers with homogenous antigen expression.

**Figure 6:**
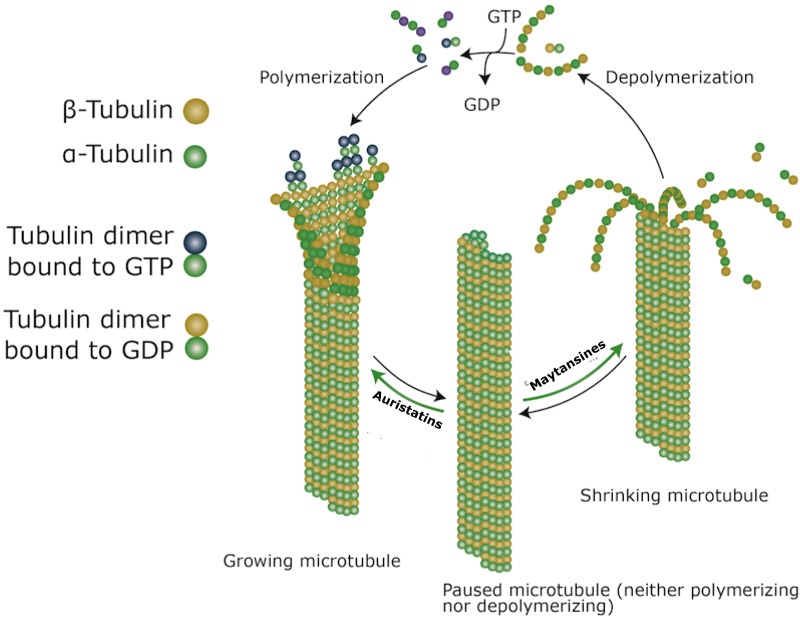
Effect of auristatins and maytansines on microtubule formation [138,173]

Similar to auristatins, maytansines, the derivatives of which are known as maytansinoids (DMs), also interfere with microtubule assembly but are mechanistically similar to vinca alkaloids [170]. Maytansines take effect by binding and capping the ‘plus’ end of the growing microtubule and block the polymerization of tubulin dimers preventing the formation of mature microtubules. Existing microtubules further disassemble once the GTP molecule on the β-subunit (of the α-β tubulin dimer) is hydrolysed, which again freezes the cell in metaphase preventing cell division (Figure 6) [171]. Of the two DMs, DM1 and DM4, the former is used as the active drug in T-DM1 [172].

Calicheamicins, duocarymycins and PBD dimers are all different types of DNA-damaging agents that are functionally similar to anthracyclines in that they all target the minor groove of DNA [174–176]. The DNA double helix forms two grooves which are present as a result of the geometric conformation of the two antiparallel strands [177]. The minor groove is narrower and consists of fewer exposed base pairs in comparison with the major groove. Calicheamicin is the extremely potent anti-tumour antibiotic used in gemtuzumab ozagamicin [178]. It binds the minor grooves of tumour cell DNA where it forms reactive diradical species that ultimately cause cleavage of the DNA strands at various locations [179]. Duocarmycins and PBD dimers take effect in similar ways with the former acting as a DNA minor groove alkylating agent and the latter as a minor groove cross-linker [180,181]. As with any normal tissue, damage to the DNA of a cancer cell induces cell death via apoptosis.

## CLINICAL DEVELOPMENT OF ADCs

Many lessons have been learnt since the FDA withdrawal of gemtuzumab ozogamicin regarding the design and development of ADC components. Pharmaceutical companies investing in ADC research have made significant advances in linker technology, conjugation chemistry, antibody engineering and the identification of potent cytotoxins, resulting in rapid evolution of the field. This has not only led to the recent approvals of brentuximab vedotin and trastuzumab emtansine but has also driven the clinical development of ADCs. Currently approved ADCs and those in advanced clinical development are listed in Table 1.

**Table 1:** ADCs in the market and in late clinical development [185] Abbreviations: DLBCL, diffuse large B-cell lymphoma; NaPi2b, sodium-dependent phosphate transport protein 2B; SPDB, disulfide N-succinimidyl 4-(2pyridyldithio)butyrate; SPP, N-succinimidyl 4-(2-pyridyldithio)pentanoate.

ADC	Sponsor	Indications	Target antigen	Antibody type	Linker	Cytotoxin	Status/Phase
Gemtuzumab ozogamicin	Pfizer	AML	CD33	Humanized IgG4	Acid-labile hydrozone 4-(4-acetylphenoxy) butanoic acid	Calicheamicin	FDA approved in 2000. Withdrawn in 2010
Brentuximab vedotin	Seattle Genetics	Relapsed Hodgkin lymphoma and systemic anaplastic large cell lymphoma	CD30	Chimeric IgG1	Cathepsin cleavable valine-citrulline	MMAE	Accelerated approval by the FDA in 2011
T-DM1	Genentech	Relapsed or chemotherapy refractory HER2-positive breast cancer	HER2	Humanized IgG1	*N*-succinimidyl 4-(*N*-maleimidomethyl) cyclohexane-1-carboxylate (SMCC)	DM1	FDA approved in 2013
Inotuzumab ozogamicin	Pfizer	Aggressive non-Hodgkin's lymphoma (stopped) Acute lymphoblastic leukaemia	CD22	Humanized IgG4	Acid-labile hydrozone (4-(4-acetylphenoxy) butanoic acid)	Calicheamicin	III
Pinatuzumab vedotin (RG-7593)	Genentech	DLBCL and follicular non-Hodgkin's lymphoma	CD22	Humanized IgG1	Cathepsin cleavable valine-citrulline	MMAE	II
RG-7596	Genentech	DLBCL and follicular non-Hodgkin's lymphoma	CD79b	Humanized IgG1	Cathepsin cleavable valine-citrulline	MMAE	II
Lifastuzumab vedotin (RG-7599)	Genentech	Non-small-cell lung cancer; ovarian tumour	NaPi2b	Humanized IgG1		MMAE	II
Glembatumumab vedotin	Celldex therapeutics	Breast cancer, melanoma	Glycoprotein NMB	Human IgG2	Cathepsin cleavable valine-citrulline	MMAE	II
Coltuximab Ravtansine (SAR-3419)	Sanofi	DLBCL; acute lymphoblastic leukaemia	CD19	Chimeric IgG1	Disulfide SPDB	DM4	II
Lorvotuzumab mertansine (IMGN-901)	ImmunoGen	Small-cell lung cancer	CD56	Humanized IgG1	Disulfide SPP	DM1	II
Indatuximab Ravtansine (BT-062)	BioTest	Multiple myeloma	CD138	Chimeric IgG	Disulfide SPDB	DM4	II
Anti-PSMA ADC	Progenics	Prostate cancer	PSMA	Human IgG1	Cathepsin cleavable valine-citrulline	MMAE	II
Labetuzumab-SN-38	Immuno-medics	Colorectal cancer	CEA (also known as CD66e)	Humanized IgG1	Lysine	Irinotecan metabolite (SN-38)	II
MLN-0264	Takeda-Millennium	Gastrointestinal tumour; solid tumours	Guanylyl cyclase C	Human IgG	Protease cleavable	MMAE	II
ABT-414	AbbVie	Glioblastoma; non-small-cell lung cancer; squamous cell tumours	EGFR			MMAF	I/II
Milatuzumab doxorubicin	Immuno-medics	Chronic lymphocytic leukaemia; multiple myeloma; non-Hodgkin's lymphoma	CD74	Humanized IgG1	Hydrazone	Doxorubicin	I/II

One particularly promising ADC, inotuzumab ozogamicin (Pfizer), has recently been discontinued for the treatment of aggressive non-Hodgkin's lymphoma following the failure of a once-a-month dose of the drug with rituximab to meet the primary objective of improving overall survival. This was in comparison with a combination of bendamustine/gemcitabine with rituximab. However, it continues to be investigated in phase 3 for acute lymphoblastic leukaemia, recognized in the U.S. and the E.U. as an orphan indication with few other treatment options [182].

Despite the strong ADC pipeline, the cost of ADC development remains a major drawback in terms of their widespread use in the clinic. The current cost of treatment with the mAb trastuzumab, is approximately $767 per dose [183], with the price almost doubling following conjugation to a cytotoxin. The production of ADCs, in particular, is especially expensive due to the incorporation of conjugation technology that complicates their manufacturing process. Research is currently directed at decreasing the costs of ADC production with the development of cheaper techniques to produce mAbs such as the use of algae as a vector for protein expression [184].

## DISCUSSION: CHALLENGES AND FUTURE OF ADCs

The major challenges associated with the development of ADCs arise from factors that interfere with ADC efficacy and/or those that result in ADC-mediated non-target cell toxicity. All three components of an ADC contribute to these challenges and need to be optimized to create a successful conjugate. Once the therapeutic effect of an ADC has been maximized, it is also desirable to prolong these effects by avoiding resistance mechanisms that decrease the duration of ADC efficacy. All targeted cancer therapies (including ADCs) are prone to resistance mechanisms that alter the function of the target antigen and render the treatment redundant [186]. The future of ADCs will mainly depend on our ability to tackle these challenges.

With more research being conducted on the use of various targeted therapies, there has been a considerable increase in the number of target antigens viable for antagonism by ADCs [187]. A selective process may therefore be employed when choosing a target that is not only widely expressed and tumour-specific but also displays minimal susceptibility to mutations and therefore to resistance. To prevent resistance and ensure the long-term use of any targeted therapy, the target antigen and the signalling pathway it triggers have to remain stable during the treatment period. Unlike traditional chemotherapeutic agents that are intrinsically non-specific and have poorly understood mechanisms of action, the targeted nature of ADCs allows better understanding of resistance mechanisms [188]. The specific type of resistance varies depending on the type of tumour antigen (EGFR, HER2) and the tumour pathway (RAS [rat sarcoma]–RAF [rapidly accelerated fibrosarcoma]–MAPK, PI3K–AKT) and can be either genetic (e.g., mutations in target antigen/downstream proteins) [189] or non-genetic (e.g., activation of compensatory signalling) [190]. Further investigation into other routes of resistance may allow the development of new drugs capable of overcoming resistance.

With regard to mAbs, lack of specificity to the target antigen might result in cytotoxic effects on healthy tissue, whereas poor internalization or inadequate antigen-binding affects the therapeutic effect of the ADC. In addition to these challenges, the large size of mAbs limits their capacity to penetrate tumour tissues, which therefore complicates the use of ADCs against solid tumours. In order to combat this, current research has explored the use of antibody fragments such as diabodies and minibodies (Figure 7) which, by virtue of their size, have better tumour penetration but shorter half-lives in comparison with standard mAbs [191,192]. The size of diabodies also allows better tumour binding as the number of antigen-binding sites per unit area is increased. Although the clinical efficacy of diabodies is yet to be demonstrated, their potential application in cancer treatment has been proven in several preclinical models [193–195]. However, as previously outlined, the current optimization criteria for ideal mAbs need not necessarily translate into improved ADCs. As more clinical data emerges, it might be possible to identify clearer principles for developing mAbs in the context of ADCs.

**Figure 7:**
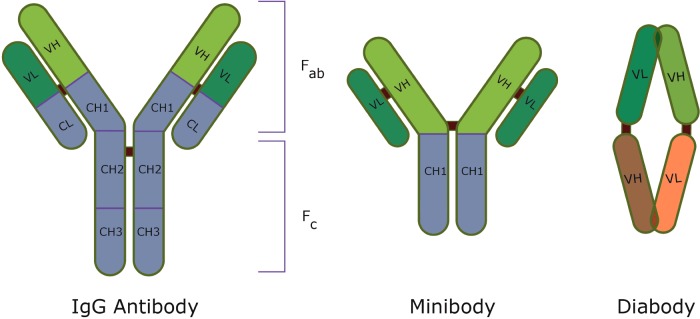
Structures of IgG antibody, minibody and diabody [196]

The future of linker chemistry is moving strongly toward the identification of new conjugation techniques that yield homogenous ADC mixtures. Research within this area has greatly expanded in the past few years with the identification of new techniques to attach cytotoxic drugs to antibodies.

Despite advances in many aspects of ADC development, the list of amenable cytotoxins for use in ADCs has remained relatively short [197]. This is an indication of the challenges involved in finding soluble, linkable drugs that are potent enough to allow for the optimal DAR of 2-4 drugs per antibody [130]. Cytotoxins such as auristatins or maytansines that selectively target rapidly replicating cells are less susceptible to non-target toxicity in case the ADC mis-delivers the drug to non-dividing cells. On the other hand, DNA-damaging agents capable of causing apoptosis in all cells are more likely to be poorly tolerated and have far more side effects [47]. Clinical trials of ADCs that utilize both types of drugs have however shown that they are commonly associated with neutropenia or thrombocytopenia [198,199] which may in some cases limit their maximum dose [200]. As more cytotoxins are being identified, it is likely that the future of ADCs will produce a diverse range of drugs with different mechanisms of action and fewer side effects [201].

Most cytotoxins are susceptible to resistance via an array of mechanisms [202]. Resistance usually occurs through the overexpression of non-specific, active transporters, such as multi-drug resistance protein (MRP) or P-gp on cancer cells [203]. These transporters sense cell-damaging agents within tumour cells and expel them. As the active sites for both P-gp and MRP are found within the cell membrane of cancer cells [204,205], cytotoxins given in the form of ADCs are less affected by cellular efflux as they are internalized once bound to their antigen. A major priority in the development of ADCs has been to further reduce the effects of active transporters and thereby increase intracellular concentrations of the cytotoxic drug. The two ways by which this can be achieved are through the administration of adjuvant drugs that block P-gp and MRP or through the conjugation of a cytotoxin that is a poor substrate for these transporters [206,207]. One research group has developed a novel hydrophilic linker, that when conjugated to maytansine, was processed by the tumour cell to form an active cytotoxic drug that was a poor substrate to P-gp [208]. Other mechanisms of resistance include decreased activation of drug, enhanced expression of drug-metabolising enzymes, increased DNA repair and failure to apoptose following drug action [209].

Despite challenges in their design, ADCs have created a new paradigm for novel cancer chemotherapy. With the specificity of mAbs and the cytotoxic capacity of small molecule drugs, ADCs promise to be a large part of the future of precision medicine as well as combination treatment. As more clinical trials are conducted on existing ADCs, it should be possible to fine-tune the components of forthcoming conjugates and improve their therapeutic risk-benefit ratio. Finding new target antigens for solid tumours, improving the understanding of mAb activity and developing novel cytotoxin-linker pairs would all pave the way for a new generation of ADCs.

## Abbreviations

ADC: antibody–drug conjugates
ADCC: antibody-dependent cellular cytotoxicity
AML: acute myeloid leukaemia
CD: cluster designation
CDC: complement-dependent cytotoxicity
CDCC: complement-dependent cell-mediated cytotoxicity
CEA: carcinoembryonic antigen
CH: constant heavy chain
DAR: drug–antibody ratio
DM: maytansinoid
EGFR: epidermal growth factor receptor
FcRn: neonatal Fc receptor
FDA: Food and Drug Administration
HER2: human epidermal growth factor receptor 2
mAb: monoclonal antibody
MAPK: mitogen-activated protein kinase
MMAE/F: monoethyl auristatin E/F
MRP: multi-drug resistance protein
NK: natural killer
P-gp: P-glycoprotein
PBD: pyrrolobenzodiazepine
PI3K: phosphoinositide 3 kinase
PSMA: prostate-specific membrane antigen
T-DM1: trastuzumab emtansine
